# An efficient and cost effective method of RNA extraction from mucilage, phenol and secondary metabolite rich bark tissue of *tossa* jute (*C. olitorius* L.) actively developing phloem fiber

**DOI:** 10.1007/s13205-016-0415-9

**Published:** 2016-04-12

**Authors:** S. B. Choudhary, M. Kumar, I. Chowdhury, R. K. Singh, S. P. Pandey, H. K. Sharma, P. G. Karmakar

**Affiliations:** 1 Central Research Institute for Jute and Allied Fibres, Barrackpore, Kolkata, WB India; 2 Indian Institute of Science Education and Research, Kolkata, WB India

**Keywords:** *Tossa* jute, Phloem fiber, Lignin, RNA, CCoAMT1

## Abstract

*Tossa* jute is an important natural fiber crop of Southeast Asian countries including India, Bangladesh, China, Thailand, Myanmar etc. Traditional industrial application of jute fiber is limited to the packaging products like hessians, sacks, etc. and the fiber found unsuitable for textile industries largely due to significantly high lignin content. Therefore, understanding genetic factors underlying lignin biosynthesis in *tossa* jute holds promise for jute based product diversification. The major limiting factor in undertaking such study is unavailability of efficient protocol for RNA extraction at secondary growth active stage of *tossa* jute. Here we report a simplified, swift and cost effective protocol for isolating fairly good quality RNA from bark tissue of 65-days-old field grown *tossa* jute plant with active secondary growth. The purity, concentration and integrity of extracted RNA ascertained. To confirm downstream amenability, isolated RNA samples were reverse transcribed and PCR analysis done by using CCoAMT1 primer. Results established that method of RNA extraction presented here is an improvement over the other methods, particularly for bark tissue of field grown *tossa* jute at advance developmental stage. Therefore, present study will enhance our ability to understand expression pattern of fiber formation and maturation related genes in mature bark tissue that holds key for much talked genetic manipulation of fiber quality via lignin optimisation in the crop.

## Introduction

*Tossa* jute (*Corchorus olitorius* L.) is an annual herbaceous dicot plant, belongs to family Malvaceae and mostly cultivated in Southeast Asian countries as a fiber crop. Besides, traditional applications in hessian and packaging industries, jute fiber valued for potential diversified industrial applications including yarn, ethanol and different grades of high quality pulp production (Rio et al. 2009). With the changing fragile climate and fast depleting natural resources their commercial prospects seems brighter than ever before.

To tap these opportunities jute fiber quality need to be improved as per industrial standards that warrant precise understanding of fiber developmental and maturation process in the crop. Isolation of pure and un-degraded RNA from *tossa* jute bark tissue actively producing secondary phloem fiber cells is the fundamental requisite for any such downstream analysis. Although, number of RNA isolation protocols developed across *planta* using either guanidinium thiocyanate or phenol/SDS (Tan and Yiap 2009) but found difficult in polysaccharides, oil and other secondary metabolites like phenolic compounds rich plants (Ghawana et al. 2011). This problem is particularly acute in case of *tossa* jute bark rich in mucilage; a highly acidic and proteinaceous compound (Stephen et al. 2006). Mucilage often binds to other secondary metabolites, co-precipitates with nucleic acids during extraction (Samanta et al. 2011) and thereby adversely affect downstream operations like gene expression analysis (Mahmood et al. 2011). Jute plants are also rich in phenolic compounds (Oboh et al. 2012) that produce quinones upon oxidization and hinder RNA isolation and/or downstream applications by binding with RNA (Loomis 1974). In addition, secondary metabolites found in the plant often co-precipitate with RNA and affect yield, quality (Bugos et al. 1995) and interfere with downstream applications (Ghawana et al. 2007). Concentration of these compounds particularly mucilage accentuated with tissue age due to formation of wide mucilage canals from surrounding mucilage cells (Kundu et al. 1959). As a result, no protocol has been described in literature to extract RNA from *tossa* jute bark tissue old enough to actively produce secondary phloem fibers. Here we report a simple, swift and cost effective protocol for isolating good quality RNA from bark tissue of 65-days-old field grown *tossa* jute plant at optimum increment percentage of phloem fiber cells.

## Materials and methods

### Plant material

*Corchorus olitorius* cv. JRO 204 seeds were soaked in distilled water for 2 h and then sown in Central Research Institute for Jute and Allied Fibers (CRIJAF), Barrackpore, India experimental field (22.45°N, 88.26°E; 3.14 above msl.) during March–July, 2014 following the recommended cultural practices. Fertilizer were applied at the rate of 40 kg N, 20 kg P_2_O_5_ and 20 kg K_2_O per hectare at sowing time, with N 50 % as basal dose and 50 % a top dress at 21 days after sowing. Adequate measures were taken to avoid abiotic and biotic stresses that may affect plant growth and phloem fiber development. The seeds were germinated and grown for 120 days in the experimental field (mean day/night temperature: 31.7/22.6 °C; RH: 65.4–89.5 %).

### Identification of jute bark developmental stage with optimum increment percentage of phloem fiber cells

Fresh free-hand transverse sections were prepared at 5 days interval from lower stem segment of *tossa* jute plants since 30 days after showing. The section was stained with safranine dye without fixation and observations were made under a Zeiss Axioskop 40 (Carl Zeiss, Jena, Germany) bright field microscope and a Canon PowerShot A80 camera system.

### Sample collection for RNA extraction

**Fig. 1:**
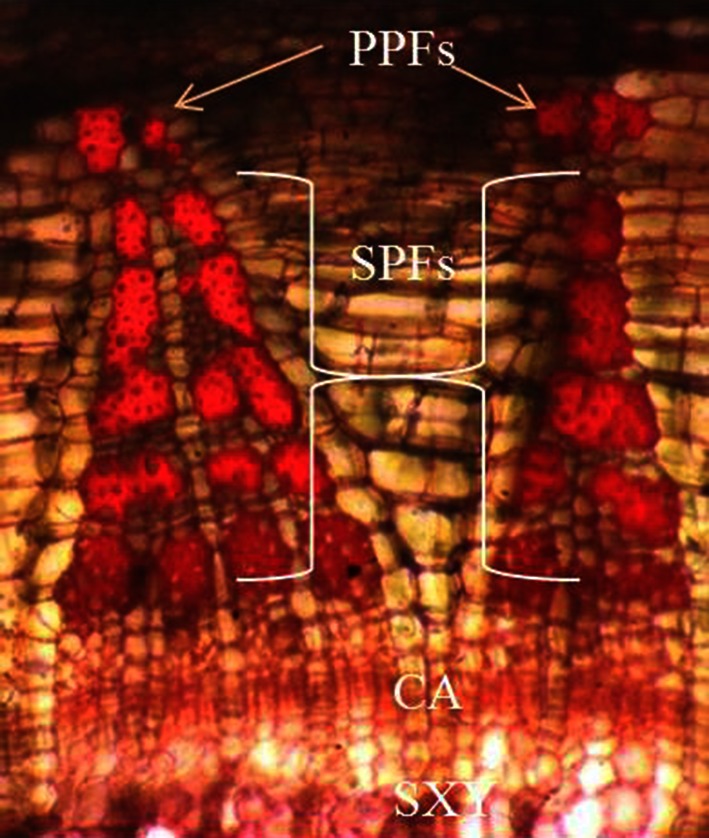
Safranin-stained transversal section (×100) of lower stem segment of *tossa* jute plant at 65 days after sowing. Triangular FCB (Fibre Cell Bundle) wedges are distinct in the lower stem segment of the plant at the stage. *Ca* cambium, *PPF* primary phloic fiber bundle, *SPF* secondary phloic fiber bundle, *SXY* secondary xylem

Bark tissue of the cultivar were taken at optimum phloem fiber cells increment percentage stage (65 days after sowing) as revealed from histological study of transverse section of basal stem (Fig. 1). Samples of the bark tissue were collected from field grown plants at base and washed with DEPC-treated water. Dried and frozen in liquid nitrogen for RNA extraction. The samples were stored at −80 °C until further use.

### RNA isolation protocol

RNA isolation protocol developed by Mahmood et al. (2011) for in vitro grown 3-days-old *Corchorus* spp. seedling was modified for effective RNA isolation from 65-days-old field grown bark tissues. 1 g of bark tissues frozen in liquid nitrogen were taken in a pre-chilled RNase-free mortar and pestle. The sample finely grounded by using liquid nitrogen through crushing and quickly divided into four aliquots. Each of them was transferred to a 2 ml RNase-free microcentrifuge tube and 1 ml of RNA extraction buffer [phenol solution equilibrated with tris HCl (pH 8.0), SDS 0.2 % (w/v), sodium acetate (3 M), EDTA (0.5 M, pH 8.0), Ambion UltraPure™ DNase/RNase-Free Distilled Water] added into each tube. The mixture was shaken vigorously for 1 min, and then kept at room temperature for 10 min, followed by addition of 0.2 ml chloroform and mixing. The content was centrifuged at 10,000 rpm for 10 min at 4 °C to carefully collect upper aqueous phase. To this, 2X volume of absolute alcohol and 0.1X volume of 5 M NaCl was added, mixed well, allowed precipitation of the pellet for 3 h. The pellet was collected following centrifugation at 12,000 rpm for 10 min at 4 °C. Initial steps of RNA extraction were repeated again after dissolving the pellet in RNase-free distilled water. 0.2 ml chloroform is added into each tube, mixed well by inversion, vortexed briefly and incubated at room temperature for 5 min to allow the nucleic acid to precipitate, followed by removal of residual phenol. The tubes were then centrifuged at 13,000 rpm for 10 min at 4 °C and the aqueous phase was transferred to a fresh micro centrifuge tube using a sterile transfer pipette. An equal volume of 7.5 M LiCl was added to each tube, mixed well by invert mixing and incubated for 10 min at room temperature to allow the nucleic acid to precipitate. The pellet was collected by centrifugation at 13,000 rpm for 10 min at 4 °C and washed twice with 0.5 ml of 70 % ethanol followed by centrifugation at 12,000 rpm for 10 min at 4 °C. The supernatant was discarded and RNA pellet dried at room temperature inside a laminar flow clean air work station. The dried RNA pellet re-suspended in 20 μl RNase-free distilled water and kept at 4 °C for immediate use or at −20 °C for long term storage.

### Assessment of RNA quantity and quality

**Fig. 2:**
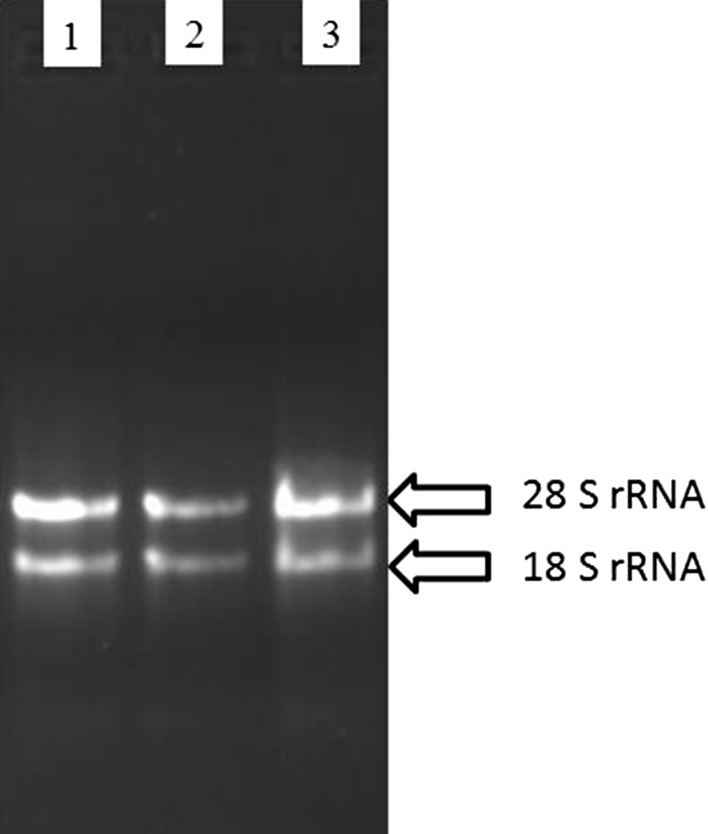
Integrity and separation of RNA samples isolated from *tossa* jute stem tissue (3 days old seedling stage) on agarose gel: **a** using modified SDS/Phenol method; **b** using SDS/Phenol method (Mahmood et al. 2011); and **c** using HBIC method (Samanta et al. 2011)

**Fig. 3:**
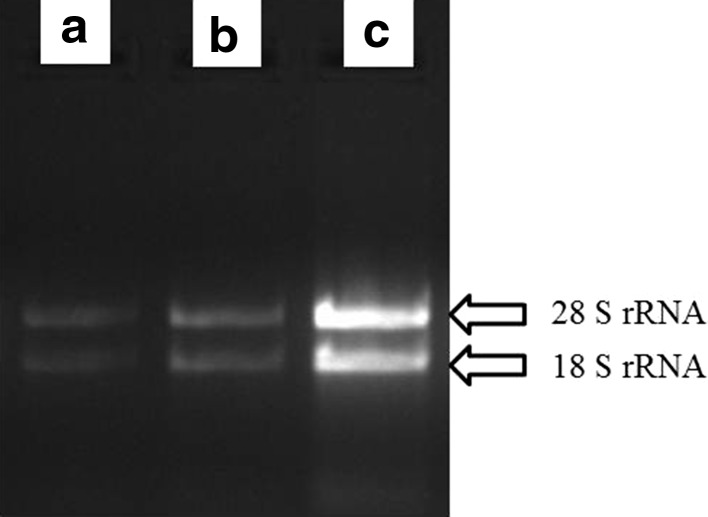
Integrity and separation of RNA samples isolated from *tossa* jute bark tissue (at 65 days after sowing) on agarose gel: **a** using SDS/Phenol method (Mahmood et al. 2011); **b** using HBIC method (Samanta et al. 2011); and **c** using modified SDS/Phenol method

Purity and concentration of RNA samples were assessed using a spectrophotometer (NanoDrop, 2000). Integrity of total RNA was evaluated on a 1 % denaturing formaldehyde-agarose gel by using 28 S rRNA and 18 S rRNA universal markers (Sambrook et al. 1989) (Figs. 2, 3).

### Reverse transcription (RT-PCR) and PCR analysis

To confirm downstream amenability, isolated RNA samples were reverse transcribed using RNA preparations (2 μg) in the presence of 1 μg oligo (dT)_12–18_ and 400 U of reverse transcriptase Superscript II (Invitrogen). Primers of two genes namely 18S rRNA gene (18s-F:5′-GTGGAGCGATTTGTCTGGTT-3′, 18s-R: 5′-TGTACAAAGGGCAGGGACGT-3′) and phenypropanoid pathway gene i.e. CCoAMT1 (F: 5′-GAGC CAGAGCCAATGAAGGA-3′, R: 5′-GTCAAGAACAGGCAAAGCAG-3′) designed (Zhang et al. 2014). PCR was performed using 1 μl of cDNA template, 0.2 mM of dNTPs, 1 U of Taq DNA Polymerase, 1× PCR buffer and 0.2 μM each primer in a final volume of 25 μl. PCR was carried out following initial denaturing template at 94 °C for 4 min. The cyclic parameters were 94 °C, 30 s; 54 °C, 40 s; 72 °C, 1 min for 35 cycles, followed by a final extension period at 72 °C for 7 min. The amplified product was checked by agarose gel electrophoresis.

## Result and discussion

The aim of this study was to develop a simple, swift and cost effective protocol for RNA isolation from mucilage, phenol and secondary metabolite rich bark tissue of *tossa* jute (*C. olitorius* L.) at optimum increment percentage of phloem fiber cells so that phloem fiber development and maturation specific gene expression study can be precisely undertaken.

### Identification of developmental stage having optimum increase in fiber cell bundles in *tossa* jute

Histological analysis (Fig. 1) suggests that at 65 days after sowing fiber cell bundles are well developed and differentiated into primary phloem fibers (PPFs) and secondary phloem fibers (SPFs). The result is in close agreement with earlier reports suggesting that the maximum phloem fiber development in jute take place during 70–84 days after sowing (Maiti and Mitra 1972; Kundu et al. 2012). Slight deviation from the earlier report with respect to developmental stage with optimum increment in fiber cell bundles may be attributed to species difference, genetic makeup and environmental conditions. Therefore, this is the ideal growth stage to study genetic regulatory mechanism underlying fiber development and lignin biosynthesis in *tossa* jute.

### RNA isolation methodology

There are very few published RNA isolation methods (Mahmood et al. 2011; Samanta et al. 2011) that could be used as reference protocols for our investigation as jute tissues are rich in complex macromolecular components. Moreover, histological evidence indicates that bark tissue of 65-days-old plant after sowing is ideal for understanding genetic regulatory mechanism of fiber development and maturation in the crop. Thus, any RNA extraction method intended to such downstream analysis must be able to extract quality RNA in good quantity from 65-days-old plant bark tissues. To this end, initially we used the protocol suggested by Mahmood et al. (2011) but unable to extract good quality RNA of desirable quantity from bark tissue of field grown *tossa* jute at 65-days-old plant. It was evident that the protocol was reported only for 3-day-old seedling plants which are obviously developmentally totally different than a 65-days-old adult plant with high amounts of mucilage as well as secondary metabolites. Next, we investigated the protocol suggested by Samanta et al. (2011) which is reported for extracting RNA from 45-day-old plant tissue from another cultivated species of the genus namely *white* jute (*C. capsularis L.*). Despite of very long and complex procedure involving 3 h centrifugation and costly chemicals like CsCl, the protocol yielded poor quality and quantity of RNA from 65-days-old plant bark. Plant growth stage and species difference may contributed to the RNA yield. However, both the protocols were able to extract good quality RNA at 3-days-old seedling stage (Table 1). Therefore, for isolating nucleic acid from *tossa* jute bark tissue at active secondary growth stage these protocols need to be modified. In this backdrop, we evaluated the protocol suggested by Mahmood et al. (2011) due to species similarity and swiftness. Mahmood et al. (2011) proposed the use of strong denaturants such as SDS and phenol that helped to remove contaminating proteins. Further, to separate RNA from DNA as well as proteins, acidic pH of extraction buffer is maintained (Chomczynski and Sacchi 1987). LiCl based RNA precipitate from nucleic acid has been employed to preferentially precipitates RNA from DNA, protein or carbohydrate solution (Barlow et al. 1963; Cathala et al. 1983; Smart and Roden 2010). In a modified protocol, we reduced the extraction volume to decrease contaminating gDNA extracted with the RNA during the LiCl precipitation step and twice purification step using extraction buffer intermittent with dissolving crude nucleic acid pellets in 1 M NaCl. Intermittent dissolution with NaCl reduce viscosity of mucilage, a major contaminant that bind to other secondary metabolites and co-precipitates with nucleic acids during extraction (Samanta et al. 2011). Such a role of NaCl has been described previously for nucleic acid extraction in mucilage rich tissue including jute (Chen and Chen 2004; Ghosh et al. 2009; Kundu et al. 2011). With reduced viscosity of mucilage in lyophilised plant tissue crude nucleic acid easily get separated during repeated washing with extraction buffer.

**Table 1:** Spectrophotometric comparison between yields and qualities of RNA samples isolated following different protocols at different growth stages

Methods	A260/280 (3 DAS)	Mean yield of RNA μg/gm of tissue (3 DAS)	A260/280 (65 DAS)	Mean yield of RNA μg/gm of tissue (65 DAS)
Phenol/SDS method (Mahmood et al. 2011)	1.86 ± 0.032	342.6 ± 9.4	1.58 ± 0.010	76 ± 4.6
HBIC method (Samanta et al. 2011)	1.94 ± 0.036	319 ± 11.5	1.63 ± 0.039	82 ± 15.68
Modified phenol/SDS method	1.96 ± 0.011	353.7 ± 8.1	1.85 ± 0.020	224.9 ± 11.24

### Electrophoresis of RNA

The yield and quality of extracted RNA from both 3-days-old stem and 65-days-old bark tissues by the present protocol were compared with other two methods by visualizing the intensity of 18S rRNA and 28 S rRNA on agarose gel and by spectrophotometric measurements. It is evident from the result that all the three protocols efficiently extracting RNA at 3-days-old growth stage but not at the later stage (Fig. 2) when concentration of mucilage and phenols increased many fold.

However, reduction of the extraction volume and twice purification step using extraction buffer intermittent with dissolving of crude nucleic acid pellet in 1 M NaCl in the present protocol efficiently eliminated most of the interfering molecules of bark tissues at older stage (65 days after sowing) and yielded good amount of RNA with better quality then HBIC method and SDS/phenol method, suggested by Samanta et al. (2011) and Mahmood et al. (2011) respectively. The extracted RNA was devoid of any DNA contamination. The ratio of *A*_260/280_ was within 1.85–1.97 signifying high purity of RNA without any contamination with polysaccharides and protein (Table 1). The sharpness and intensity of the bands of 18S rRNA and 28S rRNA transcript in agarose gel electrophoresis (Fig. 3) indicated the least degradation of isolated RNA in the proposed protocol among the methods employed.

### Validation of the present method for isolating RNA from bark tissue of *tossa* jute

**Fig. 4:**
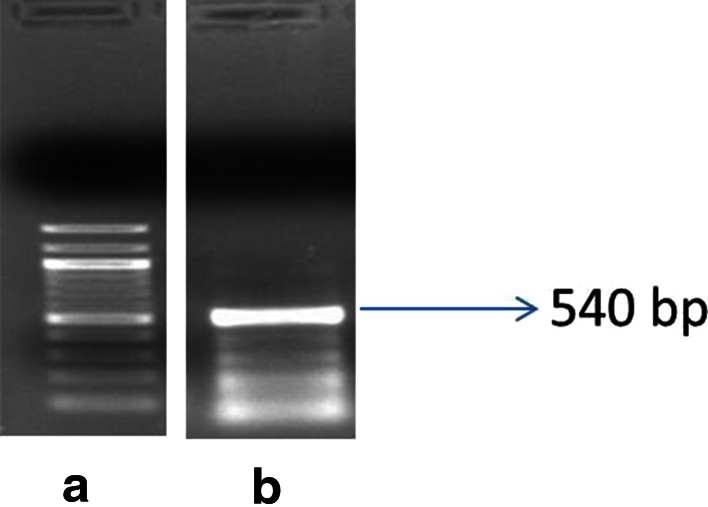
RT-PCR analysis of CCoAMT1 gene: **a** 100 bp ladder and **b** CCoAMT1

Suitability of the purified RNA for downstream applications was validated by the RT-PCR after synthesis of cDNA. First strand cDNA was synthesized from the polyA^+^ mRNA isolated from bark tissue of 65-days-old plant. RT-PCR was performed for CCoAMT1 transcript of lignin biosynthetic pathway as lignin is being considered as one of prime factor involved in fiber cell maturations (Day et al. 2005). A fragment of about 540 bp was successfully amplified from cDNA using CCoAMT1 primer described in *Corchorus capsularis* by Zhang et al. (2014) (Fig. 4).

## Conclusion

A suitable method for extraction of RNA from mature bark of *tossa* jute plant has largely been missing. Unlike to pre-existing RNA isolation protocols from secondary metabolite, phenol (Ghawana et al. 2011) and mucilage (Samanta et al. 2011) rich tissues, in the present procedure twice purification step using extraction buffer intermittent with dissolving of crude nucleic acid pellet in 1 M NaCl was used to decrease contaminating gDNA extracted with the RNA during the LiCl precipitation step. In addition, the proposed method neither employs time consuming long-duration (3-h long) centrifugation nor costly chemical like CsCl for RNA purification. Hence, this protocol is a simpler, cost effective and efficient for simultaneously processing large number of samples. To the best of our knowledge, this is the first report on RNA isolation method from *tossa* jute bark tissues with active secondary growth stage. Therefore, present study will enhance our ability to understand expression pattern of fiber formation and maturation related genes in the mature bark tissue that holds key for much talked genetic manipulation of jute fiber quality suitable for diversified products.
